# Total Small Vessel Disease Burden Predicts Functional Outcome in Patients With Acute Ischemic Stroke

**DOI:** 10.3389/fneur.2019.00808

**Published:** 2019-08-06

**Authors:** Ying-chao Huo, Qi Li, Wen-yu Zhang, Ning Zou, Rui Li, Si-yuan Huang, Hui-qi Wang, Kai-yi Song, Rong-rong Zhang, Xin-yue Qin

**Affiliations:** ^1^Department of Neurology, The First Affiliated Hospital, Chongqing Medical University, Chongqing, China; ^2^Division of Life Sciences and Medicine, Department of Neurology, The First Affiliated Hospital, University of Science and Technology of China, Hefei, China

**Keywords:** cerebral small vessel disease, magnetic resonance imaging, excellent outcome, good outcome, acute ischemic stroke

## Abstract

**Background:** Cerebral small vessel disease (SVD) is generally considered as a cause of stroke, disability, gait disturbances, vascular cognitive impairment, and dementia. The aim of this study was to investigate whether the total SVD burden can be used to predict functional outcome in patients with acute ischemic stroke.

**Methods:** From April 2017 to January 2018, consecutive patients with acute ischemic stroke who underwent baseline MRI scan were evaluated. The functional outcome was assessed using the modified Rankin Scale (mRS) at 90 days and defined as i) excellent outcome (mRS ≤ 1) and ii) good outcome (mRS ≤ 2). Brain MRI was performed and assessed for lacunes, white matter hyperintensities (WMH), and enlarged perivascular spaces (EPVS). The total SVD burden was calculated based on lacunes, WMH, and EPVS and then summed up to generate an ordinal “total SVD burden” (range 0–3). Bivariate logistic regression models were used to identify the association between SVD and functional outcome.

**Results:** A total of 416 patients were included in the final analysis; 44.0, 33.4, 19.2, and 3.4% of the patients had 0, 1, 2, and 3 features of SVD, respectively. In regard to individual SVD feature, lacunes (OR: 0.48, 95% CI: 0.32–0.71; OR: 0.49, 95% CI: 0.31–0.77) and WMH (OR: 0.53, 95% CI: 0.34–0.82; OR: 0.53, 95% CI: 0.33–0.85) were negatively associated with excellent outcome and good outcome. As to the total burden of SVD, three SVD features had strongest negative associations with functional outcomes (excellent outcome, OR: 0.13, 95% CI: 0.03–0.48; good outcome, OR: 0.18, 95% CI: 0.06–0.54). After adjustment for potential confounders, a high SVD burden (3 features, OR: 0.07, 95% CI: 0.01–0.41) and the score of total SVD burden (OR: 0.64, 95% CI: 0.44–0.93) remained negatively associated with excellent outcome.

**Conclusion:** Total SVD burden negatively associated with functional outcome at 3 months in patients with acute ischemic stroke and is superior to individual SVD feature in prediction of functional outcome. MRI-based assessment of total SVD burden is highly valuable in clinical management of stroke victims and could help guide the allocation of resources to improve outcome.

## Introduction

Cerebral small vessel disease (SVD) is prevalent in older people and generally considered as a common cause of stroke, gait disturbances, and vascular cognitive impairment (1, 2). Brain magnetic resonance imaging (MRI) is most commonly used in clinical practice for detection of SVD and also the gold standard imaging for visualization of SVD *in vivo* (3). Imaging features of SVD on MRI encompass lacunes, white matter hyperintensities (WMH), cerebral microbleeds (CMB), and enlarged perivascular spaces (EPVS) (4). Lacunes, WMH and EPVS were associated with increased risk of stroke recurrence, mortality, disability, and worse clinical outcomes (5–9). SVD is thought to be a poor prognostic marker of stroke (10).

Previous studies mainly focused on the effect of individual SVD feature on prognosis of acute ischemic stroke. Whereas, the features of SVD may occur simultaneously in a patient at clinical practice, it seems rather artificial to investigate the presence of only one feature while disregarding the others. Recently, a total SVD score has been proposed and validated (8, 11, 12), assessing the cumulative effect of different SVD features on the whole brain rather than considering individual features separately. Several recent studies have suggested a role for the total SVD score in predicting poor life quality (13), recurrent stroke (8), and mortality (14) after stroke.

However, the information about prognostic implications of the total SVD burden for 90 days functional outcome is scarce. Only two studies had reported the relationship of total SVD burden and 90 days functional outcome among patients with acute ischemic stroke (3, 7), but they just evaluated the combination effect of lacunes and WMH. Whether the total SVD burden is superior to individual SVD feature in prediction of functional outcome after stroke still needs further investigation. Understanding the factors influencing functional outcome is highly valuable in clinical management of stroke victims, and may help to guide rehabilitation strategies to improve outcomes.

Thus, the purpose of our study was to investigate the predictive efficacy of total SVD burden for functional outcome at 90 days after acute ischemic stroke. Since the evaluation of CMB is limited as susceptibility weighted imaging (SWI) and gradient recalled echo (GRE) sequence were not routine sequences of MRI for patients with acute ischemic stroke in most hospitals of China, the use of the total SVD score (11) in clinical practice is limited in China. Therefore, we only chose features of SVD (lacunes, WMH, and EPVS) that were available with routine sequence of MRI, to ensure feasibility of use and transferability of results on clinical practice.

## Materials and Methods

### Study Population

For this retrospective analysis, we used prospectively collected data from a database involving consecutive patients with acute ischemic stroke admitted between April 2017 and January 2018 at the Department of Neurology of First Affiliated Hospital of Chongqing Medical University, a large tertiary medical center in Chongqing. Chongqing is the largest municipality in Southwest China, with a population of 35 million (15). Acute ischemic stroke was diagnosed if there were new focal neurological deficits explained by relevant lesions detected on diffusion-weighted imaging (DWI) or computed tomography (CT). Patients were eligible for the study if they had cerebral MRI on admission. The exclusion criteria are as follows: 1) patients with contraindication to MRI or with poor quality of MRI; and 2) patients lack of 90-day modified Rankin Scale (mRS) score. All patients underwent standard evaluation, treatment, and rehabilitation that adhered to guidelines for ischemic stroke. The study was approved by the Ethics Committee of The First Affiliated Hospital of Chongqing Medical University, and all participants gave written, informed consent. The study protocol was performed in accordance with the Declaration of Helsinki.

### Collection of Demographic and Clinical Data

Clinical data were collected on admission through a specified questionnaire that included demographic data and the presence of vascular risk factors.

Body mass index (BMI) was calculated as weight divided by the square of height. Hypertension was defined as systolic blood pressure ≥140 mm Hg and/or diastolic blood pressure ≥90 mm Hg, or used antihypertensive drug (16). Diabetes mellitus was defined as fasting plasma glucose level ≥7.0 mmol/L, or used hypoglycemic drug/insulin injection (17). Hypercholesterolemia was defined as a low-density lipoprotein cholesterol concentration ≥3.4 mmol/L or a total cholesterol concentration ≥5.2 mmol/L, or previous diagnosis of hypercholesterolemia with current use of cholesterol-lowering medications (18). Current smoking was defined as consuming ≥1 cigarette each day or quit smoking ≤ 1 year; alcohol consumption was defined as alcohol consumption ≥8 g every week (19). The definition of coronary heart disease and previous stroke was according to the International Classification of Diseases, 9th version (20).

National Institutes of Health Stroke Scale (NIHSS) score, mRS score, and time from onset to admission were assessed at the time of initial presentation as part of the admission workup. Hospital stay was recorded at discharge. Stroke subtypes were determined based on the modified Trial of Org 10172 in Acute Stroke Treatment (TOAST) criteria: large artery atherosclerosis, cardioembolism, small vessel occlusion, other determined, or undetermined stroke (21).

### Outcome Measures

The functional outcome was measured with the mRS at 90 days and defined as i) excellent outcome (mRS ≤ 1) and ii) good outcome (mRS ≤ 2) (7, 22). We considered the mRS score as dichotomous outcome (0–1: excellent outcome vs. 2–6: disability/death; 0–2: good outcome vs. 3–6: functional dependence/death) and ordinal scale (0–6) (3, 7).

### Imaging

Brain MRI was performed on a 3.0-Tesla MRI system scanner (GE Medical Systems, Waukesha, WI, USA), and the images contained T1-weighted, T2-weighted, fluid attenuated inversion recovery (FLAIR), and DWI sequences. A stroke neurologist (Huo, YC), trained in MRI assessment and blinded to clinical data, rated all the available scans.

Lacunes were defined as rounded or ovoid lesions, >3 and <20 mm diameter, in the basal ganglia, internal capsule, centrum semiovale, or brainstem, of CSF signal intensity on T2 and FLAIR, generally with a hyperintense rim on FLAIR and no increased signal on DWI (4). WMH were diagnosed and scored by the revised version of the visual scale of Fazekas et al. (23). In the Fazekas rating scale, WMH are divided into periventricular white matter hyperintensities (PVWMH) and deep white matter hyperintensities (DWMH) according to anatomic location. PVWMH are scored as follows: none (0, no lesion), mild (1, caps or a pencil-thin lining), moderate (2, smooth halo), and severe (3, irregular lesions extending into the deep white matter). DWMH are scored as follows: none (0, no lesion), mild (1, punctuate foci), moderate (2, beginning confluent foci), and severe (3, large confluent lesions). EPVS were defined as small (<3 mm) punctate (if perpendicular to the plane of scan) or linear (if longitudinal to the plane of scan) lesions with signal intensity similar to that of cerebrospinal fluid on all sequence spaces and without a T2-hyperintense rim on FLAIR imaging (4). EPVS were counted at the level of centrum semiovale (CS) and basal ganglia (BG), respectively, with a validated four-point visual rating scale (0 = none; 1 = 1–10; 2 = 11–20; 3 = 21–40; and 4 = >40) (24). At both levels, we identified the slide in the most affected hemisphere only. Limited intrarater reliability testing (50 scans) showed a good reliability with kappa values of 0.81 for the presence of lacunes, 0.89 for PVWMH, 0.85 for DWMH, and 0.76 for EPVS.

To calculate the total SVD burden, we evaluated lacunes, WMH, and EPVS based on the ordinal scale developed by Klarenbeek et al. (11), which had been validated in several large studies (8, 12–14). A point was awarded if one or more lacunes were present, or WMH were extensive (DWMH Fazekas score 2 or 3, or PVWMH Fazekas score 3), or EPVS in BG were moderate to severe (scored 2–4), respectively. The three sub-scores were then summed up to generate a total SVD burden that ranged from 0 to 3.

### Statistical Analysis

Statistical analyses were performed by SPSS 19.0 software (IBM Corp., Armonk, NY, USA) and STATA 12.0 software (STATA Corp., College Station, Texas, USA). The total SVD burden was considered as ordinal scale (0–3), reflecting no features to all three features of SVD. Continuous variables were presented as mean ± standard deviations (SD) or as median and interquartile range (IQR) as appropriate. In univariate analyses, normally distributed continuous variables were compared with one-way analysis of variance or Student's *t*-test, and the variables not normally distributed were compared with Kruskal–Wallis *H* test or Mann–Whitney *U* test. Categorical variables were presented as percentages and were compared with Pearson's chi square test or Fisher's exact test.

Binary logistic regression was used to analyze the associations between SVD and functional outcomes (excellent outcome and good outcome). Firstly, unadjusted associations of individual SVD feature and total SVD burden with functional outcomes were analyzed. Secondly, adjusted associations between total burden of SVD and functional outcomes were analyzed. All multivariable analyses were first adjusted for age and sex (Model 1) and additionally adjusted for potential confounders (including age, sex, hypertension, diabetes mellitus, hypercholesterolemia, coronary heart disease, previous stroke, current smoking, alcohol consumption, proximal vessel occlusion, stroke subtype, baseline NIHSS, baseline mRS and hospital stay; Model 2). The results are shown as odds ratio (OR) and 95% confidence intervals (CI). A two-tailed *P* < 0.05 was considered statistically significant.

## Results

### Characteristics of the Study Population

A total of 557 patients with acute ischemic stroke were screened at baseline, among which 132 patients with contraindication to MRI or with poor quality of MRI and 9 patients lacking 90-day mRS scores were excluded. Finally, 416 patients with acute ischemic stroke were included in the final analysis. There were 278 males (66.8%) and 138 females (33.2%). The average age of the patients was 67 years (age range 19–94). Baseline characteristics of the study population stratified by burden of SVD are presented in Table 1. For SVD burden, 183 patients (44.0%) had an SVD burden of 0, showing no signs of lacunes, WMH, and EPVS; 139 patients (33.4%) had 1 feature of SVD; 80 patients (19.2%) presented with 2 features; and 14 patients (3.4%) presented with all 3 features.

**Table 1:** Baseline characteristics of study population stratified by burden of small vessel disease.

	**SVD = 0** **(*n* = 183)**	**SVD = 1** **(*n* = 139)**	**SVD = 2** **(*n* = 80)**	**SVD = 3** **(*n* = 14)**	***P-*value**
**Demographics**					
Age, years, median (IQR)	63 (54 – 69)	69 (60 – 77)	72 (64 – 80)	81 (68 – 83)	0.000
Sex, male, *n* (%)	114 (62.3)	96 (69.1)	57 (71.3)	11 (78.6)	0.334
BMI, median (IQR)	24.22 (21.94 – 25.84)	23.47 (20.96 – 25.54)	24.10 (21.26 – 25.95)	23.70 (21.61 – 25.05)	0.541
**Clinical history**					
Hypertension, *n* (%)	104 (56.8)	107 (77.0)	66 (82.5)	11 (78.6)	0.000
Diabetes mellitus, *n* (%)	63 (34.4)	73 (52.5)	41 (51.3)	6 (42.9)	0.006
Hypercholesterolemia, *n* (%)	68 (37.2)	54 (38.8)	37 (46.3)	7 (50.0)	0.461
Previous stroke, *n* (%)	24 (13.1)	38 (27.3)	22 (27.5)	3 (21.4)	0.004
Proximal vessel occlusion, *n* (%)	19 (10.4)	18 (12.9)	9 (11.3)	0 (0)	0.506
Thrombolysis, *n* (%)	14 (7.7)	13 (9.4)	5 (6.3)	1 (7.1)	0.867
Stroke subtype, *n* (%)					0.052
Large artery atherosclerosis	48 (26.2)	49 (35.3)	33 (41.3)	6 (42.9)	
Cardioembolism	38 (20.8)	27 (19.4)	8 (10.0)	3 (21.4)	
Small vessel occlusion	75 (41.0)	45 (32.4)	25 (31.3)	2 (14.3)	
Undetermined	8 (4.4)	3 (2.2)	1 (1.3)	0 (0.0)	
Other determined	14 (7.7)	15 (10.8)	13 (16.3)	3 (21.4)	
**Clinical variables**					
Baseline NIHSS, median (IQR)	3 (1 – 7)	4 (2 – 7)	4 (2 – 8)	5 (3 – 8)	0.093
Baseline mRS score, median (IQR)	0 (0 – 0)	0 (0 – 0)	0 (0 – 1)	0 (0 – 2)	0.000
90-day mRS score, median (IQR)	1 (0 – 2)	1 (1 – 2)	2 (1 – 3)	3 (2 – 4)	0.000

*SVD, small vessel disease; IQR, interquartile range; BMI, body mass index; NIHSS, National Institute of Health Stroke Scale; mRS, modified Ranking scale*.

Age, the proportion of hypertension, diabetes mellitus, and previous stroke differed significantly with increasing burden (0–3) of SVD (*p* < 0.05, Table 1). Sex, BMI, stroke subtype, and the presence of other vascular risk factors showed no differences among groups (*p* > 0.05).

### Functional Outcome

**Figure 1:**
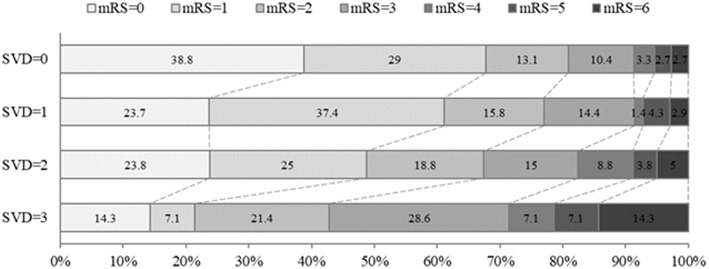
Distribution of mRS score at 90 days in all patients according to burden of small vessel disease. The percentage of participants with the mRS obtained at 90 days is shown in each cell. mRS, modified Rankin Scale; SVD, small vessel disease.

A total of 251 (60.3%) patients had excellent outcome (mRS ≤ 1) and 315 (75.7%) patients had good outcome (mRS ≤ 2) at 3 months. The incidence of excellent outcome (67.8, 61.2, 48.8, 21.4%, *p*_trend_ = 0.001) and good outcome (80.9, 77.0, 67.5, 42.9%, *p*_trend_ = 0.004) decreased significantly with increasing burden (0–3) of SVD. However, the 90-day mRS score and the proportion of 90-day mortality in patients with increasing SVD burden increased significantly (*p* < 0.001, Table 1, Figure 1).

Clinical and imaging characteristics of functional outcomes are presented in Table 2. Patients with excellent outcome or good outcome were younger and had a lower proportion of diabetes mellitus, coronary heart disease, previous stroke, and proximal vessel occlusion; a lower score of baseline NIHSS; and a shorter time of hospital stay as compared to the patients with disability/death or functional dependence/death (*p* < 0.05). In regard to stroke subtypes, 136 (32.7%) had large artery atherosclerosis, 76 (18.3%) had cardioembolism, and 147 (35.3%) had small vessel occlusion. The distribution of stroke subtypes differed significantly among all groups (*p* < 0.001, Table 2).

**Table 2:** Clinical and imaging characteristics of the study population based on functional outcomes.

	**Functional Outcomes**					
	**mRS ≤ 1** **(*n* = 251)**	**mRS ≥ 2** **(*n* = 165)**	***P-*value**	**mRS ≤ 2** **(*n* = 315)**	**mRS ≥ 3** **(*n* = 101)**	***P-*value**
**Demographics**						
Age, years, median (IQR)	65 (57 – 74)	68 (60 – 77)	0.043	66 (57 – 74)	71 (60 – 80)	0.006
Sex, male, *n* (%)	173 (68.9)	105 (63.6)	0.262	212 (67.3)	66 (65.3)	0.717
BMI, median (IQR)	23.88 (21.64 – 25.95)	24.01 (21.25 – 25.71)	0.523	24.06 (21.74 – 25.92)	23.44 (21.22 – 25.46)	0.107
**Clinical history**						
Hypertension, *n* (%)	171 (68.1)	117 (70.9)	0.548	220 (69.8)	68 (67.3)	0.634
Diabetes mellitus, *n* (%)	94 (37.5)	89 (53.9)	0.001	129 (41.0)	54 (53.5)	0.027
Hypercholesterolemia, *n* (%)	108 (43.0)	58 (35.2)	0.109	129 (41.0)	37 (36.6)	0.441
Coronary heart disease, *n* (%)	34 (13.5)	37 (22.4)	0.019	45 (14.3)	26 (25.7)	0.008
Previous stroke, *n* (%)	42 (16.7)	45 (27.3)	0.01	52 (16.5)	35 (34.7)	0.000
Current smoking, *n* (%)	106 (42.2)	67 (40.6)	0.742	135 (42.9)	38 (37.6)	0.353
Alcohol consumption, *n* (%)	68 (27.1)	32 (19.4)	0.072	81 (25.7)	19 (18.8)	0.158
Proximal vessel occlusion, *n* (%)	13 (5.2)	33 (20.0)	0.000	27 (8.6)	19 (18.8)	0.006
Thrombolysis, *n* (%)	18 (7.2)	15 (9.1)	0.478	24 (7.6)	9 (8.9)	0.676
Stroke subtype, *n* (%)			0.000			0.000
Large artery atherosclerosis	66 (26.3)	70 (42.4)		90 (28.6)	46 (45.5)	
Cardioembolism	41 (16.3)	35 (21.2)		54 (17.1)	22 (21.8)	
Small vessel occlusion	115 (45.8)	32 (19.4)		133 (42.2)	14 (13.9)	
Undetermined	6 (2.4)	6 (3.6)		8 (2.5)	4 (4.0)	
Other determined	23 (9.2)	22 (13.3)		30 (9.5)	15 (14.9)	
**Clinical variables**						
Baseline NIHSS, median (IQR)	3 (1 – 5)	7 (4 – 11)	0.000	3 (1 – 5)	8 (5 – 14)	0.000
Hospital stay, day, median (IQR)	12 (9 – 14)	15 (11 – 21)	0.000	12 (10 – 15)	15 (10 – 23)	0.000
Onset to admission time, hour, median (IQR)	17.5 (5.0 – 38.5)	15.3 (5.0 – 40.8)	0.968	17.5 (5.5 – 42.0)	11.8 (4.0 – 36.3)	0.204
**SVD**						
Lacunes, median (IQR)	0 (0 – 1)	1 (0 – 2)	0.000	0 (0 – 1)	1 (0 – 2)	0.001
WMH, median (IQR)	0 (0 – 0)	0 (0 – 1)	0.004	0 (0 – 0)	0 (0 – 1)	0.008
EPVS, median (IQR)	0 (0 – 0)	0 (0 – 0)	0.353	0 (0 – 0)	0 (0 – 0)	0.630
Total SVD burden, median (IQR)	1 (0 – 1)	1 (0 – 2)	0.000	1 (0 – 1)	1 (0 – 2)	0.003

*mRS, modified Ranking scale; IQR, interquartile range; BMI, body mass index; NIHSS, National Institute of Health Stroke Scale; SVD, small vessel disease; WMH, white matter hyperintensities; EPVS, enlarged perivascular spaces*.

**Figure 2:**
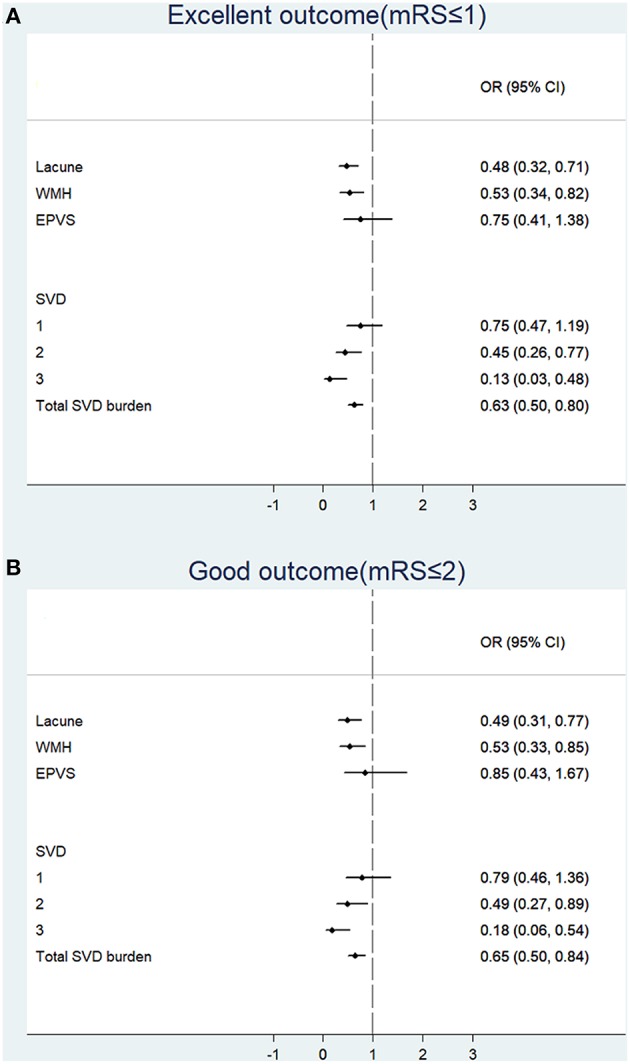
Associations of individual feature and total burden of small vessel disease with functional outcomes at 90 days. Binary logistic regression was used to analyze the associations between SVD and excellent outcome **(A)** or good outcome **(B)**. mRS, modified Ranking scale; OR, odds ratio; CI, confidence interval; WMH, white matter hyperintensities; EPVS, enlarged perivascular spaces; SVD, small vessel disease.

Logistic regression was used to analyze the associations of individual SVD feature and total burden of SVD with functional outcomes at 90 days; results are presented in Figure 2. In regard to individual SVD feature, lacunes (OR: 0.48, 95% CI: 0.32–0.71; OR: 0.49, 95% CI: 0.31–0.77) and WMH (OR: 0.53, 95% CI: 0.34–0.82; OR: 0.53, 95% CI: 0.33–0.85) were negatively associated with excellent outcome and good outcome. Further analysis of different location of WMH showed that PVWMH were associated with lower odds of excellent outcome and good outcome than DWMH (Supplementary Figure 1). As to the total burden of SVD, 2 SVD features (OR: 0.45, 95% CI: 0.26–0.77; OR: 0.49, 95% CI: 0.27–0.89), 3 SVD features (OR: 0.13, 95% CI: 0.03–0.48; OR: 0.18, 95% CI: 0.06–0.54), and the score of total SVD burden (OR: 0.63, 95% CI: 0.50–0.80; OR: 0.65, 95% CI: 0.50–0.84) were negatively associated with excellent outcome and good outcome. Among which, three SVD features had strongest negative associations with functional outcomes.

Adjusted associations between total SVD burden and functional outcomes at 90 days are presented in Table 3. After adjustment of age and sex, 2 SVD features (OR: 0.46, 95% CI: 0.26–0.82), 3 SVD features (OR: 0.13, 95% CI: 0.03–0.51), and the score of total SVD burden (OR: 0.63, 95% CI: 0.49–0.82) were negatively associated with excellent outcome; 3 SVD features (OR: 0.23, 95% CI: 0.07–0.73) and the score of total SVD burden (OR: 0.70, 95% CI: 0.53–0.93) were negatively associated with good outcome. After adjusting for age, sex, hypertension, diabetes mellitus, hypercholesterolemia, coronary heart disease, previous stroke, current smoking, alcohol consumption, proximal vessel occlusion, stroke subtype, baseline NIHSS, baseline mRS, and hospital stay, three SVD features (OR: 0.07, 95% CI: 0.01–0.41) and the score of total SVD burden (OR: 0.64, 95% CI: 0.44–0.93) remained negatively associated with excellent outcome.

**Table 3:** Adjusted associations between total burden of small vessel disease and functional outcomes at 90 days.

	**Excellent outcome** **(mRS ≤ 1) OR (95% CI)**		**Good outcome** **(mRS ≤ 2) OR (95% CI)**	
**SVD**	**Model 1**	**Model 2**	**Model 1**	**Model 2**
0	1	1	1	1
1	0.76 (0.47 – 1.23)	0.93 (0.47 – 1.85)	0.89 (0.51 – 1.57)	1.02 (0.46 – 2.29)
2	0.46 (0.26 – 0.82)	0.57 (0.25 – 1.32)	0.58 (0.31 – 1.09)	0.87 (0.34 – 2.24)
3	0.13 (0.03 – 0.51)	0.07 (0.01 – 0.41)	0.23 (0.07 – 0.73)	0.25 (0.05 – 1.20)
Total SVD burden	0.63 (0.49 – 0.82)	0.64 (0.44 – 0.93)	0.70 (0.53 – 0.93)	0.79 (0.53 – 1.19)

*Model 1: Bivariate logistic regression analyses with adjustment for age and sex*.

*Model 2: Bivariate logistic regression analyses with adjustment for age, sex, hypertension, diabetes mellitus, hypercholesterolemia, coronary heart disease, previous stroke, current smoking, alcohol consumption, proximal vessel occlusion, stroke subtype, baseline NIHSS, baseline mRS, and hospital stay*.

*mRS, modified Ranking scale; OR, odds ratio; CI, confidence interval; SVD, small vessel disease*.

## Discussion

In the present study, we found that total SVD burden (including lacunes, WMH, and EPVS) negatively affects functional outcome in patients with acute ischemic stroke and is superior to individual SVD feature in prediction of functional outcome. A high SVD burden was associated with a higher mRS score and substantially reduced the chances to have excellent outcome rather than good outcome at 90 days, indicating that a high SVD burden might predict poorer functional outcome.

In our study, lacunes and WMH were related to 90-day functional outcome, which is in accordance with previous results that lacunes and WMH negatively affect clinical outcome in patients with acute ischemic stroke. Lacunes have been reported associated with recurrent stroke and neurological impairment over time (25). Furthermore, two large-scale studies have suggested a role for WMH in predicting early neurological deterioration and disability at 3 months after stroke (6, 26). However, few studies had separately evaluated the impact of PVWMH and DWMH on functional outcome after stroke. We found that PVWMH had stronger association with functional outcome than DWMH, suggesting that PVWMH are more sensitive than DWMH in predicting poor functional outcome after stroke. Previous studies have indicated that PVWMH rather than DWMH were preferentially associated with decline in total cerebral blood flow (27), and thus may be more vulnerable to hemodynamic disturbance and more prone to ischemia owing to the unstable blood supply of the periventricular watershed area (28). It is speculated that this might be one of the reasons why PVWMH have higher efficacy than DWMH in predicting functional outcome after stroke.

We also analyzed the association between EPVS and functional outcome after stroke. EPVS, the most prevalent feature of SVD in patients with stroke (11), have been reported in relation to increased risk of recurrent ischemic stroke (9, 29). However, no independent association between EPVS and functional outcome was found in our study. Future larger multicenter studies are needed to investigate whether EPVS have a predictive effect on functional outcome in patients with acute ischemic stroke.

SVD features frequently occur together; thus, it is necessary to quantify the total burden of SVD in order to assess the cumulative effect of small vessel injury on the whole brain. We found that the total SVD burden was superior to individual SVD feature in prediction of functional outcome, with three SVD features having the strongest negative association with functional outcome. A high SVD burden (three features) was associated with higher mRS score and substantially reduced the chances to have excellent outcome rather than good outcome at 90 days. Since not all SVD features were combined in our study, the impact of total SVD burden on functional outcome might be underestimated.

The pathogenesis linking total SVD burden and functional outcome after acute ischemic stroke is not entirely clear; various mechanisms might be involved. As total SVD burden indicates the chronic accumulation of small vessel injury, which leads to the decrease of neurological reserve capacity in brain, pre-existing SVD might be a marker of increased susceptibility of brain tissue to ischemia and other injury (1, 10). Besides, a higher burden of SVD and the comorbid brain disease could destroy white matter tissue microstructure and disrupt the network architecture of the brain, thus impairing the plasticity and compensatory mechanisms and slow down the brain's recovery from stroke (30–32). What's more, SVD could affect functional outcome by disrupting motor/cognitive networks that are important for learning and neurorehabilitation (33). Evidence is accumulating that total SVD burden independently contributes to progressive cognitive impairment, dementia, and gait/balance disturbances (34–36). SVD-related cognitive/executive dysfunction may impair not only motor learning but also active participation in rehabilitation and adherence to treatment guidelines, thus leading to poor functional recovery.

Our study has several limitations. First, since the evaluation of CMB is limited as SWI and GRE sequence were not routine sequences of MRI for patients with acute ischemic stroke in most hospitals of China, we only chose SVD features that were available with routine sequence of MRI to ensure feasibility of use and transferability of results on clinical practice. As not all features of SVD were combined in our study, the impact of total SVD burden on functional outcome might be underestimated. Besides, we acknowledge that our estimation of the total SVD burden still needs further validation in larger, more varied cohorts. Second, severely affected patients who were unable to tolerate MRI and patients who underwent mechanical thrombectomy and evaluated by emergency CT examination usually lack cerebral MRI data or have incomplete MRI data, so they were excluded from the study. This selection bias would probably lead to an underestimation of the association between total SVD burden and function outcome, and might limit the generalizability of the results to all stroke patients. Third, we used qualitative (Fazekas scale) assessment for evaluation of WMH, which is considered not as precise as quantitative (volumetric) assessment. However, as qualitative and quantitative WMH burden assessments have been demonstrated to be highly correlated and comparable in stroke patients (6), quick visual rating is sufficient in WMH evaluation. Despite these limitations, the strengths of our study include relatively large sample size and apprehensive evaluation of three features of SVD with unified rating systems. More importantly, our results are easily transferrable into clinical routine.

In conclusion, our study demonstrated that total SVD burden negatively associated with functional outcome at 3 months in patients with acute ischemic stroke and is superior to individual SVD feature in prediction of functional outcome. MRI-based assessment of total SVD burden might help to identify patients with poor functional outcome. We suggest that caregivers use the knowledge of total SVD burden to provide better and more personalized care, by identifying those patients at higher risk for unfavorable functional outcome and by more clearly assessing the potential of functional recovery.

## Data Availability

The datasets generated for this study are available on request to the corresponding author.

## Ethics Statement

This study was approved by the Ethics Committee of The First Affiliated Hospital of Chongqing Medical University and all participants gave written, informed consent. The study protocol was performed in accordance with the Declaration of Helsinki.

## Author Contributions

YH and XQ helped in study concept and design. YH, WZ, NZ, SH, HW, and KS collected data. YH drafted the article. YH, QL, RL, and RZ did statistical analysis. QL, RL, and XQ did critical revision of the article. XQ obtained funding and was responsible for the administrative, technical, or material support. All authors have read and approved the final manuscript.

### Conflict of Interest Statement

The authors declare that the research was conducted in the absence of any commercial or financial relationships that could be construed as a potential conflict of interest.

## References

[1] Wardlaw JM Smith C Dichgans M. Mechanisms of sporadic cerebral small vessel disease: insights from neuroimaging. Lancet Neurol. (2013) 12:483–97. 10.1016/S1474-4422(13)70060-7 23602162 PMC3836247

[2] Pantoni L. Cerebral small vessel disease: from pathogenesis and clinical characteristics to therapeutic challenges. Lancet Neurol. (2010) 9:689–701. 10.1016/S1474-4422(10)70104-6 20610345

[3] Arba F Inzitari D Ali M Warach SJ Luby M Lees KR. Small vessel disease and clinical outcomes after IV rt-PA treatment. Acta Neurol Scand. (2017) 136:72–7. 10.1111/ane.12745 28233290

[4] Wardlaw JM Smith EE Biessels GJ Cordonnier C Fazekas F Frayne R. Neuroimaging standards for research into small vessel disease and its contribution to ageing and neurodegeneration. Lancet Neurol. (2013) 12:822–38. 10.1016/S1474-4422(13)70124-8 23867200 PMC3714437

[5] Arauz A Murillo L Cantú C Barinagarrementeria F Higuera J. Prospective study of single and multiple lacunar infarcts using magnetic resonance imaging: risk factors, recurrence, and outcome in 175 consecutive cases. Stroke. (2003) 34:2453–8. 10.1161/01.STR.0000090351.41662.91 14500936

[6] Zerna C Yu AYX Modi J Patel SK Coulter JI Smith EE. Association of white matter hyperintensities with short-term outcomes in patients with minor cerebrovascular events. Stroke. (2018) 49:919–23. 10.1161/STROKEAHA.117.017429 29540612

[7] Arba F Palumbo V Boulanger JM Pracucci G Inzitari D Buchan AM. Leukoaraiosis and lacunes are associated with poor clinical outcomes in ischemic stroke patients treated with intravenous thrombolysis. Int J Stroke. (2016) 11:62–7. 10.1177/1747493015607517 26763021

[8] Lau KK Li L Schulz U Simoni M Chan KH Ho SL. Total small vessel disease score and risk of recurrent stroke: validation in 2 large cohorts. Neurology. (2017) 88:2260–7. 10.1212/WNL.0000000000004042 28515266 PMC5567324

[9] Lau KK Li L Lovelock CE Zamboni G Chan TT Chiang MF. Clinical correlates, ethnic differences, and prognostic implications of perivascular spaces in transient ischemic attack and ischemic stroke. Stroke. (2017) 48:1470–7. 10.1161/STROKEAHA.117.016694 28495831 PMC5436733

[10] Kim BJ Lee SH. Prognostic impact of cerebral small vessel disease on stroke outcome. J Stroke. (2015) 17:101–10. 10.5853/jos.2015.17.2.101 26060797 PMC4460329

[11] Klarenbeek P van Oostenbrugge RJ Rouhl RP Knottnerus IL Staals J. Ambulatory blood pressure in patients with lacunar stroke: association with total MRI burden of cerebral small vessel disease. Stroke. (2013) 44:2995–9. 10.1161/STROKEAHA.113.002545 23982717

[12] Staals J Makin SD Doubal FN Dennis MS Wardlaw JM. Stroke subtype, vascular risk factors, and total MRI brain small-vessel disease burden. Neurology. (2014) 83:1228–34. 10.1212/WNL.0000000000000837 25165388 PMC4180484

[13] Liang Y Chen YK Deng M Mok VCT Wang DF Ungvari GS. Association of cerebral small vessel disease burden and health-related quality of life after acute ischemic stroke. Front Aging Neurosci. (2017) 9:372. 10.3389/fnagi.2017.00372 29180960 PMC5693845

[14] Song TJ Kim J Song D Yoo J Lee HS Kim YJ. Total cerebral small-vessel disease score is associated with mortality during follow-up after acute ischemic stroke. J Clin Neurol. (2017) 13:187–95. 10.3988/jcn.2017.13.2.187 28406586 PMC5392462

[15] Zhou R Zhou H Cui M Wang Y Tan J Sawmiller D. Association between aortic calcification and the risk of osteoporosis in a Chinese cohort: the chongqing osteoporosis study. Calcified Tissue Int. (2013) 93:419–25. 10.1007/s00223-013-9776-9 23975213

[16] Chobanian AV Bakris GL Black HR Cushman WC Green LA Izzo JL Jr. The seventh report of the joint National committee on prevention, detection, evaluation, and treatment of high blood pressure: the JNC 7 report. JAMA. (2003) 289:2560–72. 10.1001/jama.289.19.2560 12748199

[17] Alberti KG Zimmet PZ. Definition, diagnosis and classification of diabetes mellitus and its complications. Part 1: diagnosis and classification of diabetes mellitus provisional report of a WHO consultation. Diabet Med.(1998) 15:539–53. 10.1002/(SICI)1096-9136(199807)15:7<539::AID-DIA668>3.0.CO;2-S 9686693

[18] Expert Panel on Detection Evaluation and Treatment of High Blood Cholesterol in Adults Executive Summary of the third report of the National cholesterol education program (NCEP) expert panel on detection, evaluation, and treatment of high blood cholesterol in adults (adult treatment panel III). JAMA. (2001) 285:2486–97. 10.1001/jama.285.19.2486 11368702

[19] Zhou S Zhou R Zhong T Li R Tan J Zhou H. Association of smoking and alcohol drinking with dementia risk among elderly men in China. Curr Alzheimer Res. (2014) 11:899–907. 10.2174/1567205011666141001123356 25274108 PMC4428477

[20] Slee VN. The International classification of diseases: ninth revision (ICD-9). Ann Intern Med. (1978) 88:424–6. 10.7326/0003-4819-88-3-424 629506

[21] Adams HP Jr Bendixen BH Kappelle LJ Biller J Love BB Gordon DL. Classification of subtype of acute ischemic stroke: definitions for use in a multicenter clinical trial: TOAST: trial of org 10172 in acute stroke treatment. Stroke. (1993) 24:35–41. 10.1161/01.str.24.1.35 7678184

[22] Maestrini I Strbian D Gautier S Haapaniemi E Moulin S Sairanen T. Higher neutrophil counts before thrombolysis for cerebral ischemia predict worse outcomes. Neurology. (2015) 85:1408–16. 10.1212/WNL.0000000000002029 26362283 PMC4626239

[23] Fazekas F Chawluk JB Alavi A Hurtig HI Zimmerman RA. MR signal abnormalities at 1.5 T in Alzheimer's dementia and normal aging. AJR Am J Roentgenol. (1987) 149:351–6. 10.2214/ajr.149.2.351 3496763

[24] Potter GM Chappell FM Morris Z Wardlaw JM. Cerebral perivascular spaces visible on magnetic resonance imaging: development of a qualitative rating scale and its observer reliability. Cerebrovasc Dis. (2015) 39:224–31. 10.1159/000375153 25823458 PMC4386144

[25] van Dijk AC Fonville S Zadi T van Hattem AM Saiedie G Koudstaal PJ. Association between arterial calcifications and nonlacunar and lacunar ischemic strokes. Stroke. (2014) 45:728–33. 10.1161/STROKEAHA.113.003197 24457294

[26] Ryu WS Woo SH Schellingerhout D Jang MU Park KJ Hong KS. Stroke outcomes are worse with larger leukoaraiosis volumes. Brain. (2017) 140:158–70. 10.1093/brain/aww259 28008000 PMC6276917

[27] ten Dam VH van den Heuvel DM de Craen AJ Bollen EL Murray HM Westendorp RG Decline in total cerebral blood flow is linked with increase in periventricular but not deep white matter hyperintensities. Radiology. (2007) 243:198–203. 10.1148/radiol.2431052111 17329688

[28] Kim KW Macfall JR Payne ME. Classification of white matter lesions on magnetic resonance imaging in elderly persons. Biol Psychiatry. (2008) 64:273–80. 10.1016/j.biopsych.2008.03.024 18471801 PMC2593803

[29] Yang H Shen R Jin Z Li J Wu Y Xu Y. Dilated Virchow–Robin spaces in first-ever lacunar stroke patients: Topography and clinical correlations. J Stroke Cerebrovasc Dis. (2016) 25:306–11. 10.1016/j.jstrokecerebrovasdis.2015.09.034 26521169

[30] Helenius J Mayasi Y Henninger N. White matter hyperintensity lesion burden is associated with the infarct volume and 90-day outcome in small subcortical infarcts. Acta Neurol Scand. (2017) 135:585–92. 10.1111/ane.12670 27573379 PMC5332524

[31] Kim HJ Im K Kwon H Lee JM Kim C Kim YJ. Clinical effect of white matter network disruption related to amyloid and small vessel disease. Neurology. (2015) 85:63–70. 10.1212/WNL.0000000000001705 26062629 PMC4501945

[32] de Groot M Ikram MA Akoudad S Krestin GP Hofman A van der Lugt A. Tract-specific white matter degeneration in aging: the rotterdam study. Alzheimers Dement. (2015) 11:321–30. 10.1016/j.jalz.2014.06.011 25217294

[33] Valdes Hernandez Mdel C Booth T Murray C Gow AJ Penke L Morris Z. Brain white matter damage in aging and cognitive ability in youth and older age. Neurobiol Aging. (2013) 34:2740–7. 10.1016/j.neurobiolaging.2013.05.032 23850341 PMC3898072

[34] Liu R Chen H Qin R Gu Y Chen X Zou J. The altered reconfiguration pattern of brain modular architecture regulates cognitive function in cerebral small vessel disease. Front Neurol. (2019) 10:324. 10.3389/fneur.2019.00324 31024423 PMC6461194

[35] Jiang Y Wang Y Yuan Z Xu K Zhang K Zhu Z. Total cerebral small vessel disease burden is related to worse performance on the Mini-Mental State Examination and incident dementia: A prospective 5-year follow-up. J Alzheimers Dis. (2019) 69:253–62. 10.3233/JAD-181135 31006685

[36] van der Holst HM Tuladhar AM Zerbi V van Uden IWM de Laat KF van Leijsen EMC. White matter changes and gait decline in cerebral small vessel disease. Neuroimage Clin. (2018) 17:731–8. 10.1016/j.nicl.2017.12.007 29270357 PMC5730123

