# Supplementary material for: Total Small Vessel Disease Burden Predicts Functional Outcome in Patients With Acute Ischemic Stroke
Source: Front Neurol. 2019 Aug 6;10:808. doi: 10.3389/fneur.2019.00808 (PMC6691043; doi:10.3389/fneur.2019.00808)
Supplement: Supplementary file 1 [file Table_1.DOCX]

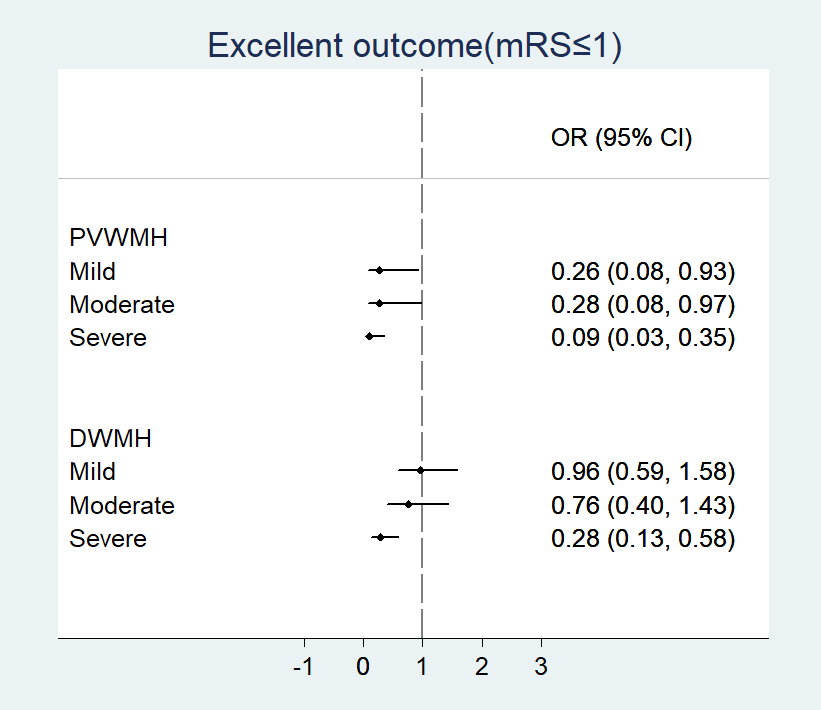

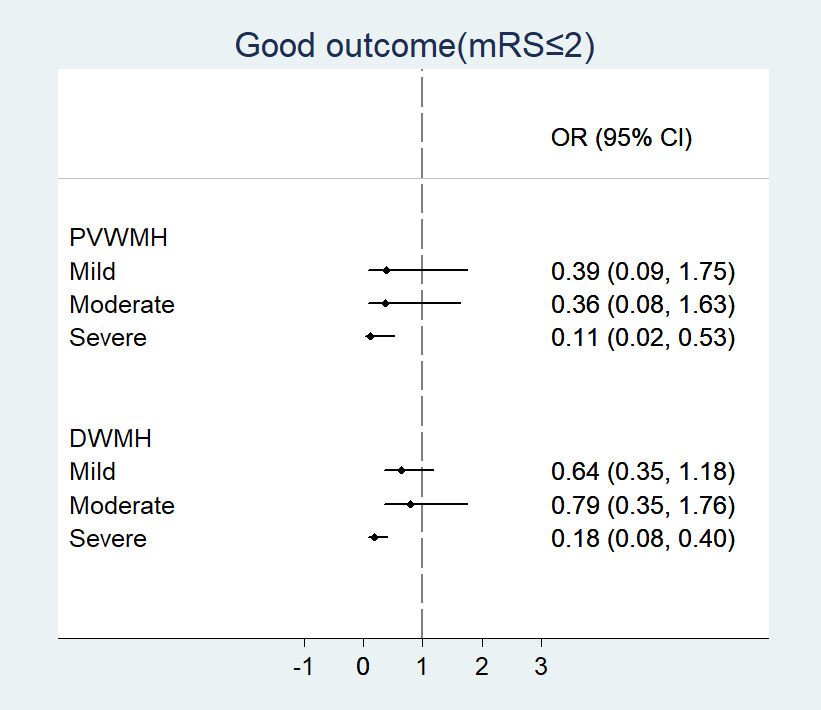


A

B

FIGURE Ⅰ. Associations between white matter hyperintensities and functional outcomes at 90 days. Binary logistic regression was used to analyze the associations between PVWMH, DWMH and excellent outcome (A) or good outcome (B).

Abbreviations: mRS, modified Ranking scale; OR, odds ratio; CI, confidence interval; PVWMH, periventricular white matter hyperintensities; DWMH, deep white matter hyperintensities.
